# Therapeutical Measures to Control Airway Tolerance in Asthma and Lung Cancer

**DOI:** 10.3389/fimmu.2012.00216

**Published:** 2012-07-26

**Authors:** Katerina Andreev, Anna Graser, Anja Maier, Stephanie Mousset, Susetta Finotto

**Affiliations:** ^1^Laboratory of Cellular and Molecular Lung Immunology, Institute of Molecular Pneumology, Friedrich-Alexander-Universität Erlangen-NürnbergErlangen, Germany

**Keywords:** allergic asthma, lung tumor, TGF-β, IL-10, IL-17A, Foxp3

## Abstract

Airway tolerance is a specialized immunological surveillance which is activated by the cells of the lung to deal with and distinguish between innocuous and pathogenic inhalants. However, this distinction does not always occur. Airway tolerance is necessary to avoid the development of allergic disorders, such as asthma, which is dominated by a pathological expansion of Th2 and Th17 cells in the airways. By contrast, tumor cells induce tolerogenic factors in their microenvironment to evade T-cell mediated anti-tumor-immune responses. This review updates current understandings on the effect of the cytokines TGF-β, IL-10, and IL-17A on the lung immune responses to antigen, and analyzes their involvement in allergic asthma and lung cancer. The aim of the review is to evaluate where therapeutic intervention may be feasible and where it might fail. The multifunctional role of these cytokines further complicates the decision on the timing and concentration for their use as therapeutical targets. In fact, TGF-β has suppressive activity in early tumorigenesis, but may become tumor-promoting in the later stages of the disease. This dual behavior is sometimes due to changes in the cellular target of TGF-β, and to the expansion of the induced (i)-Tregs. Similarly, IL-17A has been found to elicit pro- as well as anti-tumor properties. Thus, this pro-inflammatory cytokine induces the production of IL-6 which interferes with Treg development. Yet IL-17A could promote tumor growth in conjunction with IL-6-dependent activation of Stat3. Thus, understanding the mechanisms of airway tolerance could help to improve the therapy to both, allergic asthma and lung cancer. Hereby, asthma therapy aims to induce and maintain tolerance to inhaled allergens and therapy against lung cancer tries to inhibit the tolerogenic response surrounding the tumor.

## Introduction

The lung consists of two main components with distinct functions and different immunological properties: the conducting airways and the lung parenchyma. While the gas exchange takes place in the lung parenchyma within its alveoli, the conducting airways are covered with an epithelium skilled to perform a clearance of inhaled antigens (Holt et al., 2008). The parenchyma lung surface, by contrast, must be thin and permeable to allow an efficient gas exchange. However, this permeability also causes a vulnerability to infection (Brandtzaeg, 2009; Kohlmeier and Woodland, 2009). As a result, the lung immune system must manage to protect the host against pathogens, while maintaining tolerance to innocuous antigens (Holt et al., 2008).

Immunological tolerance is generally defined as the unresponsiveness of the immune system to a certain antigen, mainly a self-antigen or a harmless environmental antigen (Akbari et al., 2001). A matter of special importance in diseases is the peripheral tolerance to innocuous antigens that reach the respiratory tract by inhalation, which is referred to as airway tolerance or respiratory tolerance (Akbari et al., 2001). Immunological tolerance is generally indispensable to avoid overshooting responses to pathogens and to prevent immune responses to self-antigens.

The immunological state of the lung is usually characterized by a general hyporesponsiveness with a lack of functional allergen-specific CD4^+^ T cells, down-regulation of the IgE production and suppression of the development of airway hyper-responsiveness (AHR) and inflammation (Holt et al., 2008). However, in predisposed individuals, an exposure of the lung immune system to an antigen and its subsequent presentation by professional antigen presenting cells (APC) causes activation and pathological expansion of subtypes of CD4^+^ T helper cells. In healthy individuals, antigen presentation at first leads to activation and expansion of CD4^+^ T cells as well. However, this is followed by removal and inactivation of those antigen-specific T cells (Akbari et al., 2001). Moreover, in subjects with a primary or secondary immunodeficiency or patients with tumor, respiratory tolerance could result in worsening of the disease.

This review primarily deals with the role of airway tolerance for two typical lung diseases, namely allergic asthma and lung cancer. Although these diseases are substantially different from each other, they both seem to be associated with a dys-regulation of the tolerogenic processes in the lung. Immunological parameters, which play a role for the induction of airway tolerance, are inhibited in asthma patients and are up-regulated in lung cancer patients. Consequently, there are parallels concerning the molecular factors serving as potential targets for a therapeutic approach to both diseases, although the general aim of asthma therapy is an enhancement of airway tolerance, whereas anti-cancer immune responses require circumvention of immunological tolerance. According to that, we chose the three cytokines, transforming growth factor β (TGF-β), IL-10, and IL-17A for further discussion, focusing on their effects on asthma as well as on lung tumor and their relevance as possible therapeutic targets.

## Induction of T Cell Mediated Airway Tolerance

**Figure 1:**
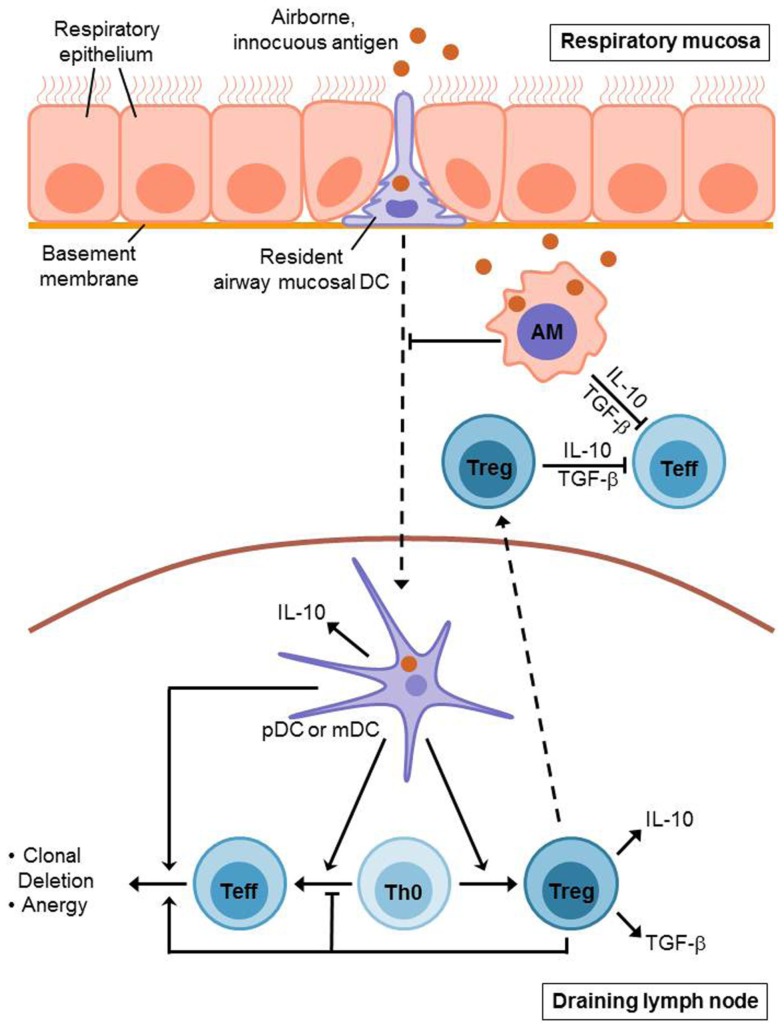
**Induction of T cell mediated airway tolerance**. Innocuous antigen reaches the respiratory epithelium where it is recognized by resident airway mucosal dendritic cells (AMDCs). These DCs extend long protrusions which enable them to sample the antigen directly from the airway lumen. Afterwards, the AMDCs (including pDCs and mDCs) migrate to the draining lymph nodes presenting the sampled antigen. Antigen presentation by mDCs leads to an initial activation and expansion of antigen-specific T cells. However, this is followed by inactivation and deletion of these effector T (Teff) cells and the generation of regulatory T (Treg) cells. This process also involves pDCs which contribute to the induction of Treg cells as well. Treg cells mediate clonal deletion and anergy of Teff cells in the draining lymph nodes as well as in the respiratory mucosa where they are recruited to during tolerance induction. Alveolar macrophages (AMs) contribute to airway tolerance via phagocytosis and sequestration of incoming antigens. Moreover, they directly inhibit T cell responses and seem to suppress dendritic cell migration as well as antigen presentation. Treg cells, AMs, and pulmonary DCs each secrete immunosuppressive cytokines such as IL-10 and TGF-β.

During the induction of airway tolerance, an initial exposure of the airways to an innocuous antigen leads to activation and expansion of antigen-specific T cells which is followed by the recruitment of regulatory T cells (Tregs) to the lung mucosa and a subsequent suppression of antigen-specific immune responses. Further recognition of the same antigen by the lung immune system does not lead to another effector cell expansion, but causes the induction of an antigen-specific T cell tolerance (Figure 1). This process is suggested to be primarily T cell-mediated, whereas Forkhead box P3 (Foxp3)^+^ CD4^+^ CD25^+^ Tregs are considered to play a prominent role for the underlying immunosuppressive mechanisms (Holt et al., 2008). There is a distinction between thymus-derived naturally arising Foxp3^+^ regulatory T cells and Tregs, which are induced from naïve T cells and can acquire Foxp3 expression in response to specific tolerogenic stimuli, such as TGF-β or retinoic acid (RA). Aside from that, there are other T cell subtypes, which do not necessarily express Foxp3, but still exhibit regulatory function, as for example Tr1 and Th3 cells (Sakaguchi et al., 2008). Generally, Tregs represent a crucial factor for the regulation of peripheral T cell responses in virtually all immunological fields. They are able to mediate clonal deletion and anergy of effector T cells as well as suppression of effector functions (Fontenot et al., 2003; Roncarolo et al., 2006; Tang and Bluestone, 2008). Several mechanisms are proposed to mediate Treg-dependent immunosuppression, as for example cell-contact dependent inhibition and secretion of immunosuppressive cytokines, such as IL-10 or TGF-β (Ostroukhova et al., 2004; Holt et al., 2008; Sakaguchi et al., 2008). However, the exact mechanisms underlying airway tolerance induction are still unclear. Noteworthy, animal studies demonstrated that Treg-mediated maintenance of airway tolerance seems to require continuous exposure to airborne antigens, as antigen withdrawal results in a decreased number of Tregs, going along with an enhanced susceptibility to inappropriate Th2 cell dependent immune reactions against respiratory antigens (Holt et al., 2008).

Prior to the recruitment of Tregs to the respiratory mucosa and the induction of T cell tolerance, the immune system of the lung must recognize the incoming antigens and ensure that they are not pathogenic. This process requires the interaction of innate and adaptive immunity, whereas the identification of potentially pathogenic antigens is facilitated through pattern recognition receptors, such as Toll-like receptors (TLRs) and involves the action of pulmonary dendritic cells (Holt et al., 2008). The conducting airways of the lung contain dense networks of specialized dendritic cells underneath and within the epithelium, comprising both myeloid DCs (mDCs) and plasmacytoid DCs (pDCs). These so-called resident airway mucosal DCs (AMDCs) extend long protrusions into the airway lumen through the intact epithelium to directly sample inhaled antigens (de Heer et al., 2005; Holt et al., 2008). Under non-inflammatory conditions, the main function of AMDCs consists in a continuous uptake of inhaled antigens and a subsequent migration to regional lymph nodes (RLNs), where the harmless antigens are presented in a tolerogenic manner (de Heer et al., 2005; Holt et al., 2008). Noteworthy, AMDCs lack the ability to efficiently present antigens, as long as they reside in the lung mucosa, however they develop this capability after the migration to the RLNs (Holt et al., 2008). mDCs and pDCs both seem to contribute to the mechanisms which are involved in airway tolerance induction. Thus, the presentation of innocuous antigens by mDCs induces activation and proliferation of antigen-specific naïve T cells, however, instead of antigen-specific immunity this causes T cell tolerance. This could possibly be explained by an incomplete activation of mDCs under anti-inflammatory conditions resulting in an abortive immune response, which means that either the antigen-specific T cells are deleted or that Tregs are generated instead of effector T cells (de Heer et al., 2005). In contrast to that, the tolerogenic role of pDCs is primarily based on their ability to induce the generation of Tregs. Moreover, pDCs have been shown to produce indoleamine 2,3-dioxygenase (IDO), which mediates inhibition of T cell proliferation. Finally, it has also been demonstrated that airway tolerance induction is associated with an increased production of IL-10 by lung DCs leading to suppression of effector cell responses in the airways (de Heer et al., 2005).

Beside Tregs and dendritic cells, alveolar macrophages also contribute to the maintenance of immunological homeostasis in the lung. In contrast to AMDCs, alveolar macrophages do not migrate to the RLNs. Instead, the primary function of alveolar macrophages during airway tolerance induction is the prevention of adaptive immune responses mediated by phagocytosis and sequestration of incoming antigens. Thus, alveolar macrophages are suggested to directly inhibit T cell responses, using inhibitory mediators, such as IL-10, TGF-β, prostaglandins, or nitric oxide. However, there is also evidence that alveolar macrophages have an influence on dendritic cell number and function, suppressing antigen presentation as well as the ability of DCs to migrate to RLNs (Holt et al., 2008).

The following paragraphs describe the molecular factors which are involved in airway tolerance induction as well as their influence on the development of allergic asthma and lung cancer. Considering the current knowledge on the mechanisms of respiratory tolerance induction, the immunosuppressive cytokines TGF-β and IL-10 might be of particular importance and will therefore be discussed in more detail. The third cytokine, which will be focused on in this review, is IL-17A. In contrast to TGF-β and IL-10 it is a pro-inflammatory cytokine and its role in airway tolerance induction still needs to be elucidated. However, there is increasing evidence that IL-17A plays an important role in the pathogenesis of allergic asthma and lung cancer.

## T Cell Mediated Allergic Asthma

Asthma is a chronic inflammatory disease of the airways and is characterized by chronic airway inflammation, increased mucus production, reversible airway obstruction, remodeling of the airways, and AHR. More than 300 million people worldwide suffer from this disease and the number of affected people grows steadily.

To date, asthma is considered not-curable, however, there are options to control this disease. On one hand asthma therapy consists of so-called “controllers” such as long-acting β_2_-agonists and steroids that need to be taken regularly to alleviate the symptoms and beware of or rather delay the exacerbation of asthma, depending on the degree of the disease. On the other hand there are therapeutics, known as “relievers”, including short-acting β_2_-agonists which are used for the treatment of acute asthma attacks to achieve an immediate bronchodilatation (Ukena, 2008). Nevertheless, the major goal of asthma research remains to find a way to cure this disease. There are various forms of asthma, including allergic asthma, asthma induced by exposure to air pollution or cigarette smoke and severe steroid-resistant asthma, also known as allergic, non-allergic, and intrinsic asthma (Kim et al., 2010). In this review we will focus on the most common form of asthma: allergic asthma.

In healthy subjects, the respiratory confrontation with an innocuous antigen first leads to a short-lived induction of a local immune response to this antigen, followed by long-term peripheral tolerance (Lowrey et al., 1998). In asthmatic patients, harmless antigens can provoke an unwanted Th2 sensitization to these aeroallergens and cause Th2 responses (van Rijt et al., 2005). Allergic asthma therefore has been found to be characterized by a pathological expansion of at least Th2 cells and by the lack of T regulatory cells. One hypothesis states that this could be due to a lack of early childhood-exposure to infectious agents increasing the susceptibility to allergic diseases by suppressing natural development of the immune system (Umetsu et al., 2002). Th2 cells and their cytokines are responsible for the recruitment of eosinophils to the airways (IL-5) and for the allergen-specific development of IgE (IL-4) (Umetsu et al., 2002). Furthermore, IL-9 and IL-13 have recently been found to be involved in the pathogenesis of allergic asthma (Holgate and Polosa, 2008; Kim et al., 2010). It is also known that lung DCs are necessary for the development of Th2 cells during the establishment of airway inflammation seen in allergic asthma (Kim et al., 2010). In contrast to that, Th1 cells are suggested to exhibit a regulatory function in the context of allergic asthma as IFN-γ suppresses the differentiation of Th2 cells. Thus, investigators show great interest in the Th1/Th2 balance to find new therapeutic modalities in asthma (Park and Lee, 2010). Th17 cells are the third subset of T helper cells which are also suggested to be relevant for the development of asthma, although its particular role is not completely clear and requires further investigation.

**Figure 2:**
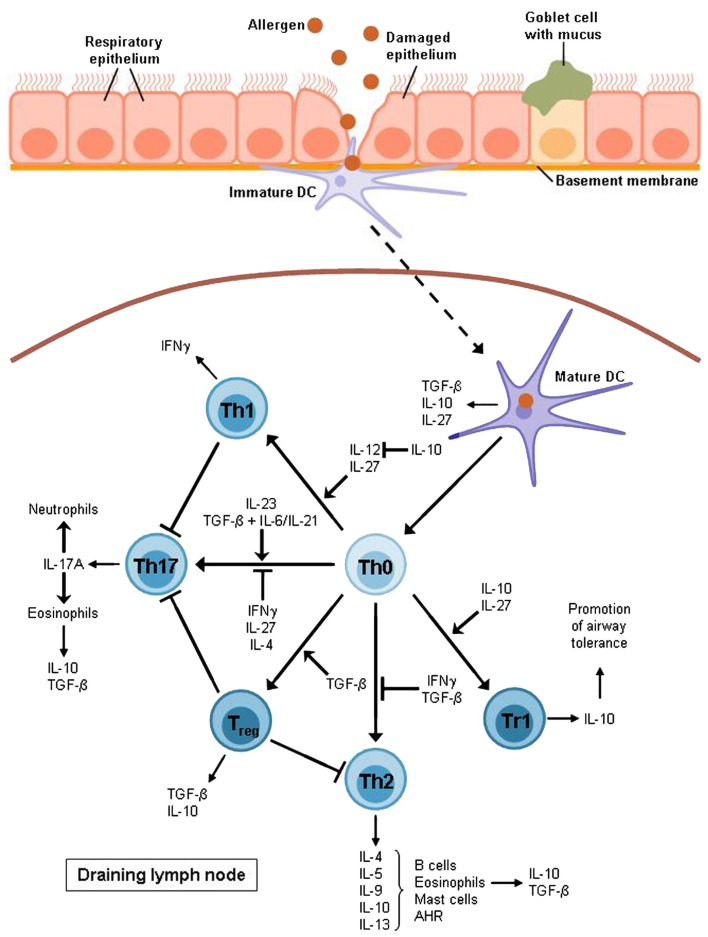
**T cell differentiation in allergic asthma**. After allergen uptake the DC migrates to the lymph node where it activates naïve T cells to develop into different effector subsets. Allergic asthma has been found to be characterized mostly by Th2 cell expansion and a lack of Treg cells. Th2 cytokines raise the production of allergen-specific immunoglobulin E (IL-4), support the growth of eosinophils (IL-5) and mast cells (IL-9) and directly cause airway hyperreactivity (AHR; IL-13). Furthermore, Th2 cells produce IL-10 which is also expressed by eosinophils, DCs, and Treg cells, in particular Tr1 cells which also require this cytokine for differentiation. Non-asthmatic airways show a balance between Th1 and Th2 cells as IL-10 is able to suppress the differentiation of the Th1 subset by blocking IL-12 synthesis whereas the Th1 cytokine IFNγ contributes to the inhibition of Th2 development. However, this balance is impaired in allergic asthma. The Th1 subset is also supposed to have an inhibitory function on Th17 cells which produce mainly IL-17A leading to the recruitment of eosinophils and neutrophils to the airways. The development of Th17 cells depends on IL-23 or TGF-β combined with either IL-6 or IL-21.TGF-β is produced by eosinophils, DCs, and Treg cells whereas it is also required for the induction of Treg cells and for the inhibition of the Th2 subset. Apart from IL-10 and TGF-β, DCs also produce IL-27, an inhibitor of Th17 cells and an inducer of Tr1 cell development. Except for DCs, this Treg subset is also supposed to be involved in the induction of airway tolerance.

The overall objective of asthma research is to find a possibility to inhibit the exaggerated immune response found in asthmatic patients. Hereby, airway tolerance inducing agents serve as promising targets for a potential vaccination strategy as airway tolerance is suggested to protect against and control the onset of asthma by inducing T regulatory cytokines and other mediators (Neurath et al., 2002). There are two main suppressor cytokines that are released by regulatory T cells and concomitantly are involved in their induction. As shown in Figure 2 these two important cytokines released by Tregs control the pathological expansion of the Th2 and Th17 cells resulting in inhibition of the downstream inflammatory response observed in allergic asthma.

### The role of transforming growth factor-β in allergic asthma

The profibrotic cytokine TGF-β exists in three highly homological isoforms, TGF-β1, TGF-β2, and TGF-β3 (Halwani et al., 2011), binding to the same receptor complex (Miyazono et al., 1994). TGF-β is secreted as an inactive homodimer bound to a latent complex (Makinde et al., 2007) which is targeted to the extracellular matrix in association with TGF-β binding proteins. The final activation of the cytokine is achieved by several proteases, including α_v_β_6_ integrin, MMp-2, and -9, plasmin, thrombospondin-1, and calpains (Makinde et al., 2007). According to Halwani et al. (2011) also retinoids, tissue transglutaminases, reactive oxygen species, and a low pH are involved in the TGF-β activation.

Transforming growth factor-β and other members of this family are believed to be involved in the initiation, maintenance, and resolution of inflammatory responses (Halwani et al., 2011). Their importance in maintaining immune homeostasis has been demonstrated using TGF-β knockout mice which exhibited multifocal inflammatory lesions, especially in lungs and hearts, and died within the first weeks of life (Shull et al., 1992; Kulkarni et al., 1993).

Accordingly, TGF-β seems to play an important role for the development of asthma as asthmatic patients show high levels of TGF-β in the bronchoalveolar lavage fluid (BALF).

The cytokine is expressed and secreted in the lung by nearly all structural immune cells as well as by inflammatory cells that are recruited to the lung during asthma exacerbation. However, eosinophils are believed to be the main source of TGF-β in asthmatic airways (Ohno et al., 1992) and may play an important role in airway remodeling. Halwani et al. (2011) demonstrated that in severe asthma 65% of TGF-β1 mRNA positive cells were eosinophils and that 75% of eosinophils were TGF-β1 positive. In non-asthmatic individuals the main TGF-β source is the airway epithelium, but also fibroblasts, endothelial cells, vascular and airway smooth muscle cells were reported to produce TGF-β.

T cells were identified as the central effector cells in TGF-β mediated regulation of airway responses (Schramm et al., 2003). TGF-β has an immunoregulatory role via its direct suppression of T cell proliferation and macrophage activation. Over-expression of TGF-β in T cells resulted in the suppression of allergic asthma in a murine asthma model (Halwani et al., 2011). Moreover, adoptive transfer of Tregs over-expressing TGF-β were able to confer complete resistance towards the induction of antigen dependent airway hyperreactivity only in the presence of IL-10 indicating an additive immunosuppressive role of these two cytokines (Presser et al., 2008). In contrast, impairment of TGF-β signaling led to increased allergic airway responses in transgenic mouse models compared to wild-type mice (Schramm et al., 2003; Presser et al., 2008).

One central role of T cells in airway inflammation is the production of Th2 cytokines such as IL-4, IL-5, and IL-13. The latter was increased in the BALF and sera of the Tg CD2-DNTGF-βRII mice in a murine model of allergic asthma (Walter et al., 2001; Schramm et al., 2003). These data indicate an inhibitory role of TGF-β on IL-13 and consequently on AHR in a setting of allergic asthma. Besides its anti-inflammatory effect, TGF-β is also a pro-inflammatory cytokine. It is involved in the airway remodeling process in asthma and other inflammatory and immune-mediated lung diseases (Halwani et al., 2011). Furthermore, it exhibits antagonistic effects on epithelial cells. In fact, it either protects epithelial cells from apoptosis through the Smad2/3 pathway or it induces an apoptotic effect on these cells by activating the p38 mitogen-activated protein kinase (MAPK) signaling pathway. The p38 MAPK pathway is activated upon stress or in response to chemical agents (Makinde et al., 2007) and it is usually associated with cell survival and proliferation. However, its activation in response to TGF-β promotes cell apoptosis (Makinde et al., 2007) which results in the detachment of epithelial cells from the basement membrane (Halwani et al., 2011). The increased epithelial damage facilitates the development of an asthmatic phenotype. TGF-β is also able to enhance the Fas-induced apoptotic and fibrotic effect in alveolar epithelial cells. However, in the central airway epithelial cells TGF-β induces an inhibitory effect on Fas-induced apoptosis (Makinde et al., 2007). Additionally, the expression of TGF-β2 by bronchial epithelial cells after challenge with IL-13 results in an increased formation of mucin. Treatment with an antibody against TGF-β caused a decrease in the number of mucus secreting goblet cells in an asthmatic mouse model (Makinde et al., 2007). Furthermore, mucus production and secretion is enhanced in fibroblasts due to an increased expression of IL-6 induced by TGF-β (Makinde et al., 2007). However, the expression of pro-inflammatory TGF-β is resistant to the effects of corticosteroids (Halwani et al., 2011). Taken together, these data indicate an anti-inflammatory function of TGF-β when targeting T cells and a pro-inflammatory function on airway epithelial cells and fibroblasts. Up to now, there exists no satisfying treatment opportunity for allergic asthma. One of the aims of allergen-specific immunotherapy is the induction of peripheral T cell tolerance, which is characterized by the generation of allergen-specific Treg cells. Released by Tregs, TGF-β has an anti-inflammatory effect. Besides inhibiting B-cell proliferation and differentiation, TGF-β decreases immunoglobulins excluding mucosal IgA (Fujita et al., 2012). However, Presser et al. (2008) suggested that the suppressing capacity of TGF-beta overproducing Tregs on AHR is due to the concurrent release of IL-10, not only TGF-β.

### The role of interleukin-10 (IL-10) in allergic asthma

There is increasing evidence that IL-10-producing pulmonary DCs play a very important role in airway tolerance. Akbari et al. (2001) could already demonstrate that the adoptive transfer of DCs from IL-10^−/−^ mice could not induce OVA-specific T cell unresponsiveness in recipient mice. IL-10 is an anti-inflammatory and immunosuppressive cytokine with pleiotropic effects in immunoregulation and inflammation. It is produced by various cells such as B lymphocytes, NK cells, mast cells, eosinophils, DCs, monocytes, macrophages, Tregs, and T lymphocytes. Among the T lymphocytes, Th2 cells seem to be the main producers (Hofmann et al., 2012). Its role as immunomodulatory cytokine involves the inhibition of major histocompatibility complex (MHC) class II expression, the reduction of CD80/CD86 mediated co-stimulation as well as the down-regulation of IL-1β, IL-6, IL-8, TNF-α, and IL-12 production. Thus, three crucial functions of specific and non-specific immunity, which are mediated by monocytes and macrophages, namely antigen presentation, expression of immune mediators, and phagocytosis are affected by IL-10 leading to the inhibition of airway inflammation (Sabat, 2010; Hofmann et al., 2012).

Accordingly, reduced levels of IL-10 have been found in the lungs of asthmatic patients. Furthermore, IL-10 deficient mice express higher levels of IL-4, IL-5, and IFN-γ compared to wild-type littermates leading to the suggestion that normal levels of IL-10 in healthy persons might be responsible for reduced Th2-like immune responses (Umetsu and DeKruyff, 1999). However, the role of IL-10 in allergic asthma is not clear. On one hand it is thought to reduce AHR and inflammation on the other hand it is suggested to be a crucial Th2 cytokine. Thus, IL-10 is able to suppress the production of Th1 cytokines by blocking IL-12 synthesis (Umetsu et al., 2002). In addition, IL-10 is crucial for the Th2-polarized responses in asthma and has a regulatory role in the later immune responses by down-modulating the inflammation caused by Th2 cell signaling as mentioned above (Umetsu et al., 2002). Taking the mentioned findings together, the conclusion arises that IL-10 plays an important role in asthma and airway tolerance.

Pulmonary DCs are crucial for the maintenance of airway tolerance. For instance, they produce IL-10 after uptake of harmless antigens, which is suggested to be involved in induction of Tregs, in particular T regulatory type 1 (Tr1) cells. Akbari et al. could confirm this statement by the adoptive transfer of IL-10 deficient DCs (Akbari et al., 2001; Kushwah and Hu, 2011) in a murine model of allergic asthma. The importance of IL-10-producing DCs and Tr1 cells in the maintenance of airway tolerance could also be demonstrated by an antibody-mediated blockade of IL-10 signaling (Gravano and Vignali, 2011).

Apart from IL-10, also IL-27 and TGF-β1 produced by pulmonary DCs as well as ICOS/ICOS-L signaling seem to be involved in the induction of Tr1 cells. In this term, the function of IL-27 is the stimulation of naïve T cells to express c-maf, IL-21, and ICOS. Furthermore, IL-27 activates STAT1 and STAT3 and thus drives IL-10 production in T cells (Murugaiyan et al., 2009; Pot et al., 2009; Iyer et al., 2010; Kushwah and Hu, 2011). Examination of the blood of allergic and healthy donors indicated a down-regulation of antigen-specific Tr1 cells and an up-regulation of IL-4 producing Th2 cells in allergic patients. By comparison, in healthy subjects IL-10-producing Tr1 cells are the predominant antigen-reactive T cell population (Umetsu et al., 2002; Hammad and Lambrecht, 2008). Thus, the induction of Tr1 cells and therefore IL-10 might be a new therapeutical aim in the treatment of allergic asthma since Tr1 cells inhibit airway hyperresponsiveness, amongst other features of allergic asthma (Akbari et al., 2001; Umetsu et al., 2002).

### The role of interleukin-17 (IL-17) in allergic asthma

Recently, Th mediated immunity has enlarged to include a third subset of effector helper T cells, the Th17 cells, termed after IL-17A, their preferentially produced cytokine (Park and Lee, 2010; Aujla and Alcorn, 2011). Beside Th17 cells, other cells such as eosinophils, NK cells, neutrophils, NKT cells, and γδ T cells also express IL-17A (Korn et al., 2009). It is already known that TGF-β, a profibrotic cytokine which is also crucial for airway tolerance, in combination with IL-6 or IL-21 drive the differentiation of Th17 cells and therefore the production of the pro-inflammatory cytokine IL-17A by inducing RORγt that is thought to be the master regulator of Th17 cells. TGF-β in conjunction with IL-6 also initiates the expression of IL-23 that in turn stimulates IL-17A production. After binding to its receptor, IL-17A signals through two different pathways, one of which is Act-1-dependent whereas the second one is Act-1-independent. The Act-1-dependent pathway includes intracellular signaling molecules such as TRAF6, TRAF3, and TAK1 as well as members of the MAP kinase family such as ERK and p38 leading to the secretion of neutrophil-mobilizing molecules. The Act-1-independent one includes JAK1 and PI3K and results in gene activation, cytokine secretion, and inactivation of GSK-3β (Ivanov and Linden, 2009).

Several studies have shown that IL-17A is up-regulated in lung tissues, BALF, sputum, and peripheral blood from patients with allergic asthma. In addition, increased levels of IL-17A mRNA were detected in the sputum of asthmatic patients where IL-17A levels correlate with the number of neutrophils (Bullens et al., 2006; Park and Lee, 2010). It has been shown that IL-17A causes neutrophilic inflammation in allergic asthma via IL-8 as both IL-17A and IL-8 mRNA are increased in the sputum of allergic patients. Furthermore, IL-17A could enhance the development of neutrophils by inducing the release of IL-6 from human bronchial fibroblasts (Park and Lee, 2010; Aujla and Alcorn, 2011). An up-regulation of IL-17A also seems to be linked to bronchial hyper-responsiveness in asthmatic patients. However, the influence of Th17 cells on the AHR is not yet completely clear as there are different findings on this aspect in murine models of allergic asthma (Park and Lee, 2010; Aujla and Alcorn, 2011).

Apart from the contribution to neutrophilic inflammation in asthma, IL-17A is also suggested to be responsible for the eosinophilia observed in the airways of asthmatic patients. It is assumed that IL-17A is able to synergize with IL-4 and IL-13 to increase Th2 cytokines and CCL11 secretion (Aujla and Alcorn, 2011). Chemokine (C–C motif) ligand 11 (CCL11) belongs to the CC chemokine family that is also known as eotaxin-1. CCL11 selectively recruits eosinophils by inducing their chemotaxis, and therefore is implicated in allergic responses (Jose et al., 1994; Garcia-Zepeda et al., 1996). It is obvious that the therapy of asthma tend to eliminate inflammatory cells such as neutrophils and eosinophils. As mentioned before, IL-17A seems to be involved in the establishment and course of asthma and may offer a new therapeutic target in the treatment of asthma.

Accordingly, blockade of IL-17A via anti-IL-17A antibody leads to a down-regulation of IL-4, IL-5, and IL-13 as well as reduced neutrophils and eosinophils in BALF as well as extenuated AHR in an allergic model of this disease (Hellings et al., 2003; Anderson, 2008; Park and Lee, 2010). Furthermore, splenocytes of IL-17RA deficient mice cultured with IL-25, a cytokine regulating allergen-induced Th2 responses and AHR, lose their ability to produce IL-5 or IL-13 (Ballantyne et al., 2007; Kuestner et al., 2007; Rickel et al., 2008; Park and Lee, 2010).

Other possibilities to target the IL-17A mediated effects are the modulation of upstream (e.g., IL-6, IL-23) or downstream mediators (e.g., MAP kinases) of IL-17A. It is known that IL-23 is needed for the development and stabilization of Th17 cells and therefore the IL-17A expression. Wakashin et al. (2008) could demonstrate that it is possible to suppress the recruitment of lymphocytes, eosinophils, and neutrophils after allergen sensitization using an antibody against IL-23-p19. In addition, a down-regulation of Th2 cytokines in murine lungs has been observed after OVA-sensitization (Wakashin et al., 2008; Ivanov and Linden, 2009; Park and Lee, 2010).

As mentioned before, another way to reduce IL-17A expression is the blockade of downstream messengers. Some groups could already show that the blockade of p38 kinase and ERK kinase results in a decreased expression of IL-6 and IL-8 from human bronchial epithelial cells. These experiments also disclosed a more potent effect of the p38 kinase inhibitor indicating that this pathway is more promising as target (Ivanov and Linden, 2009).

Taken together, there are several alternatives to regulate IL-17A expression representing new therapeutic strategies in the treatment of asthma.

## Tolerance and Lung Cancer

Lung cancer is the most common cancer-related cause of death worldwide (van Klaveren, 2009). Despite many years of research, there is still no efficient therapy against this disease. Since lung cancer is described as almost symptom free during its early stages, a large proportion of the patients already shows metastases by the time of diagnosis. As a consequence, only 15% of the patients survive for more than 5 years after primary diagnosis (van Klaveren, 2009; Reddy et al., 2011). Generally, lung cancer is thought to arise upon a number of pre-neoplastic lesions in the airway mucosa. There are two main types of lung cancer, namely small cell lung cancer (SCLC) and non-small cell lung cancer (NSCLC), whereas NSCLC is more common accounting for approximately 75% of all lung cancer cases (Ellis, 2012). NSCLC in turn involves several subtypes such as adenocarcinoma (Ad), bronchoalveolar carcinoma (BAC), squamous cell carcinoma (SCC), large cell carcinoma (LCC) as well as some mixed subtypes (Kerr, 2001; Ellis, 2012).

The predominant cause of lung cancer is tobacco smoking explaining the fact that there is a higher incidence of this disease in developed countries (van Klaveren, 2009). However, an increased lung cancer risk is also associated with inherited features as well as exposure to various environmental carcinogens such as asbestos, arsenic, radon, and polycyclic aromatic hydrocarbons (Ellis, 2012). Apart from that, there is a close relationship between the development of several kind of tumors and inflammation. However, the relationship between cancer and the immune system is ambiguous. On one hand inflammation is associated with production and secretion of tumor growth promoting molecules such as DNA-damaging agents as well as particular cytokines and growth factors, which are able to enhance cell proliferation. In accordance, chronic inflammation potentiates the risk of tumor development (Muller and Scherle, 2006). Thus, patients with chronic obstructive pulmonary disease have been shown to have a higher risk of developing lung cancer (Reddy et al., 2011).

On the other hand inflammatory immune responses are suggested to play a major role for tumor rejection. For instance, immune-compromised Rag^−/−^ and STAT1^−/−^ mice show a significantly higher incidence of tumor development (Mapara and Sykes, 2004; Muller and Scherle, 2006). Although normally the immune system is able to recognize particular tumor antigens, for example mutated epitopes, cancer cell antigens are always self-antigens, which strongly hampers the recognition of tumor cells by the immune system (Perales et al., 2002). Moreover, tumor cells have evolved numerous strategies to escape immune-mediated rejection. For example, they may lack tumor-specific antigens or co-stimulatory signals which are necessary to elicit an adequate immune response. They also might show a reduced expression of MHC class I, thereby avoiding recognition by CD8^+^ cytotoxic T cells (CTLs). Finally, tumors seem to be able to actively achieve immunosuppression by the production of anti-inflammatory molecules such as the cytokines TGF-β and IL-10, the enzyme indoleamine 2,3-dioxygenase (IDO) or the inhibitory cell-surface protein programmed death ligand-1 (PD-L1) (Byrne et al., 2011). Besides, a general immunological hyporesponsiveness and thus a suppressive microenvironment as it exists in airway tissues could make an efficient anti-tumor response even more difficult compared to other tissues. This could probably potentiate the risk of tumor growth in the respiratory tract resulting in diseases such as lung tumor (Karabon et al., 2011). Taken together, this indicates that immunological tolerance, which normally serves to protect the host from dangerous self-directed immune reactions, becomes a problem in connection with cancer diseases. For that reason, current approaches to cancer therapy are often aimed at breaking self-tolerance, thereby enhancing the anti-tumor immune response (Perales et al., 2002).

**Figure 3:**
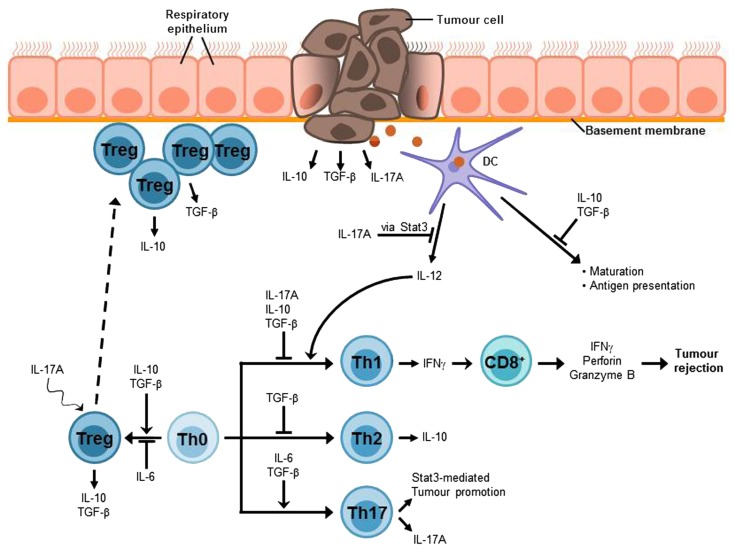
**Lung cancer-associated immunosuppressive microenvironment**. An efficient anti-tumor immune response strongly depends on IFNγ-producing Th1 cells which in turn mediate the activation of tumor-specific CD8^+^ cytotoxic T cells (CTLs) which are required for the elimination of cancer cells. The lung immune system is characterized by a general hyporesponsiveness and a suppressive microenvironment inhibiting an efficient anti-tumor immune response. This involves, *inter alia*, Treg cells, which have been found to accumulate at the tumor site and the cytokines IL-10, TGF-β, and IL-17A. These cytokines can also be produced by the tumor cells. Thus, it is possible that tumor cells contribute to the induction of Treg differentiation or at least to the recruitment of Treg cells to the tumor site. TGF-β suppresses T cell proliferation and differentiation of naïve T cells into effector memory cells as well as antigen presentation. Moreover, it is involved in the development and function of induced Treg cells. The immunosuppressive functions of IL-10 include the inhibition of Th1 cell proliferation and cytokine production as well as the suppression of antigen presentation. In addition, IL-10 is involved in Treg development and function, in particular Tr1 cells. The tumor-promoting role of the Th17 cytokine IL-17A can be attributed to its relation to the proto-oncogene Stat3. One function of Stat3 is the inhibition of IL-12 expression which is required for the induction of Th1 differentiation and thus anti-tumor immune responses. The differentiation of Th17 cells requires the presence of IL-6 and TGF-β, whereas Treg development is induced by TGF-β alone and IL-6 promotes Treg inhibition. Nevertheless, IL-17A seems to be linked to Treg development in tumor via a mechanism that is still unclear.

The most important mediators of airway tolerance are suggested to be Tregs which are suggested to hamper immune surveillance and to inhibit efficient immune responses against cancer (Sakaguchi et al., 2008). Thus, it has been shown that at early stages of cancer Foxp3^+^ Tregs accumulate at the tumor site (Figure 3; Sakaguchi et al., 2008; Byrne et al., 2011). Moreover, there is an increased number of Tregs in the peripheral blood of patients with NSCLC as well as other kinds of cancer diseases (Li et al., 2011; Onishi et al., 2012). An increased ratio of Tregs to effector T cells at the tumor site seems to correlate with poor prognosis for cancer patients (Byrne et al., 2011; Onishi et al., 2012). Based on these assumptions, numerous animal studies aimed to analyze the effects of Treg depletion or an alteration of Treg function. The results of these studies indicate that the elimination or a reduction of Tregs can break immunological tolerance to tumor cells *in vivo* and *in vitro* and induce an effective tumor-specific immune response (Shimizu et al., 1999; Sakaguchi et al., 2008). For example, elimination of Tregs and a concomitant stimulation of effector T cells resulted in tumor rejection in 90% of sarcoma-bearing mice (Whelan et al., 2010). In addition, the attempt to remove Tregs in cancer patients led to a regression of melanoma metastases (Rasku et al., 2008). According to that, Tregs represent an obstacle for successful immunotherapy against cancer (Byrne et al., 2011; Onishi et al., 2012). Therefore, a promising target for future cancer immunotherapy is to overcome Treg-mediated tumor cell tolerance. Although up to now the mechanisms of how Tregs inhibit anti-tumor responses and why Tregs accumulate at tumor sites have to be still elucidated (Li et al., 2011), there is evidence for some Treg-associated molecules to be involved in these processes such as TGF-β_1_ or IL-10. Moreover, recent data indicates that IL-17A, a molecule previously described as a pro-inflammatory factor, unexpectedly might also be connected to Treg mediated tumor promotion (Li et al., 2011; Reppert et al., 2011; Onishi et al., 2012).

### The role of transforming growth factor-β in lung cancer

Transforming growth factor-β plays a central role for the regulation of the balance between inflammation and tolerance in both alveoli and the conducting airways of the lung. TGF-β influences T cell proliferation and differentiation as well as T cell apoptosis and antigen presentation (Cottrez and Groux, 2001). In particular, this cytokine suppresses the differentiation of naïve T cells into effector memory cells and inhibits the proliferation of T cells. Moreover, it is involved in development and function of induced Tregs (iTregs). Thus, TGF-β exhibits typical features of immunosuppressive cytokines indicating a tumor-promoting role of TGF-β. However, besides its various immune regulatory functions, TGF-β is also able to inhibit epithelial proliferation and to induce expression of extracellular matrix components suggesting that TGF-β might rather act as a tumor suppressor, inhibiting the development and progression of cancer (Figure 3; Markowitz and Roberts, 1996).

Previous studies concerning the role of TGF-β in lung cancer revealed that lung cancer patients show increased serum levels of TGF-β as compared to healthy individuals (Hasegawa et al., 2001). Moreover, it has been shown that different kinds of tumor cells, including small- as well as NSCLC cells, over-express TGF-β (Wojtowicz-Praga, 2003; Jeon and Jen, 2010). Furthermore, various types of cancers have been shown to require TGF-β activity to form metastases (Roberts and Wakefield, 2003). These findings indicate that TGF-β indeed supports tumorigenesis. However, it has also been shown that higher levels of TGF-β in patients with lung Ad are associated with better prognosis (Inoue et al., 1995).

This contradiction could possibly be explained by the fact that the increased expression and activation of the TGF-β ligand during carcinogenesis is often accompanied by a decreased expression or inactivation of the TGF-β receptors resulting in an unresponsiveness of the tumor cells to TGF-β-induced growth inhibition (Roberts and Wakefield, 2003; Jeon and Jen, 2010). Thus, it has been reported that numerous tumor types are characterized by the loss of functional RII or RI TGF-β receptor due to somatic mutations (Markowitz and Roberts, 1996). The consequence could be that the role of TGF-β for cancer development changes in the course of tumorigenesis. Whereas it exhibits tumor suppressor activity at early stages of tumor development, it loses this function due to TGF-β receptor unresponsiveness and becomes a tumor promoter in late-stage disease supporting tumor invasiveness and metastases (Roberts and Wakefield, 2003; Jeon and Jen, 2010). Consistent with this idea, studies on mammary tumors in mice revealed that enhanced TGF-β signaling leads to a delayed development of primary tumors but an increased formation of lung metastases whereas a disruption of TGF-β signaling has the opposite effect leading to earlier appearance of primary tumors but a lower number of metastatic foci (Roberts and Wakefield, 2003). In consideration of these facts, the disruption of TGF-β mediated immunosuppression could be a promising therapeutic approach to cancer.

### The role of interleukin-10 (IL-10) in lung cancer

Interleukin-10 (IL-10) is a Th2 cytokine which has long been associated with anti-proliferative properties. Its immunosuppressive functions include the inhibition of Th1 cell proliferation and cytokine production, the inhibition of antigen presentation and natural killer cell activity as well as the down-regulation of tumoricidal molecules (de Vita et al., 2000; Hatanaka et al., 2000; Teng et al., 2011). In addition, IL-10 has been shown to be necessary to induce the development of T regulatory 1 cells (Tr1) *in vitro* and to be involved in the regulation of TGF-β responses, thus supporting the suppressive effects of TGF-β (Groux et al., 1997; Cottrez and Groux, 2001). A wide range of cell types is known to produce IL-10, as for example macrophages [in particular tumor-associated macrophages (TAMs)], B cells, T cells (especially Tregs), and epithelial cells (Ouyang et al., 2011; Wang et al., 2011). Besides that, IL-10 is also known to be produced and secreted by different types of cancer cells, including lung cancer (de Vita et al., 2000; Hatanaka et al., 2000; Mocellin et al., 2005).

These findings indicate that IL-10 could be involved in tumor immunosuppression. To prove this assumption, numerous studies have been performed to further analyze IL-10 regarding lung cancer. Thus, it could be demonstrated that NSCLC patients show significantly elevated IL-10 mRNA as well as serum levels as compared to healthy controls which has been shown to be associated with poorer prognosis (Hatanaka et al., 2000). Furthermore, metastatic cancer has been associated with higher IL-10 levels than cancer without metastases. In addition, there seems to be a relation between IL-10 levels and therapeutic success as a comparison of IL-10 levels in lung cancer patients treated with either radiotherapy or chemotherapy revealed that IL-10 values were significantly increased in non-responders, whereas they were decreased in responders (Wojciechowska-Lacka et al., 1996; de Vita et al., 2000; Hatanaka et al., 2000). Another study revealed that NSCLC patients with late-stage disease (stage II, III, and IV) show increased levels of TAM-derived IL-10 which is accompanied by lymph node metastases, pleural invasion, and lympho-vascular invasion (Figure 3; Wang et al., 2011).

Nevertheless, data concerning the role of IL-10 for tumor progression are partially inconsistent. Thus, in contrast to the above-mentioned results, some preclinical and clinical studies suggest that IL-10 is important for tumor rejection. Accordingly, there are indications that IL-10 expression is reduced in patients with NSCLC and that this reduction could correlate with poor prognosis (Lu et al., 2004; Mocellin et al., 2005). However, up to now a connection between higher IL-10 levels and better survival could not be shown (Teng et al., 2011). Despite these contradictions, the immune-suppressive molecule IL-10 is an important factor during the induction of airway tolerance and might be a promising target for future approaches to lung cancer therapy. Therefore, it is necessary to further define the role of IL-10 in lung cancer.

### The role of interleukin-17 (IL-17) in lung cancer

The role of IL-17A in cancer is controversial. On the one hand IL-17A has pro-inflammatory functions. Thus, IL-17A recruits neutrophils and induces the production of IL-6, IL-8, tumor necrosis factor-α (TNF-α), and IL-1β (Weaver et al., 2007). In contrast to that, most of the cytokines which are assumed to promote tumor development are anti-inflammatory cytokines, as for example IL-10 or TGF-β, indicating that IL-17A might be important for tumor rejection (Wang et al., 2009; Chen et al., 2010). According to that, tumor growth has been shown to be increased in IL-17^−/−^ mice in case of MC38 sarcoma (Kryczek et al., 2009). Nevertheless, there is growing evidence that IL-17A could rather act as a tumor promoter. For instance, an increased number of IL-17A producing cells have been detected in different types of cancer (Chen et al., 2010). Furthermore, in patients with lung Ad the mRNA levels of IL-17A as well as of the Th17 transcription factors RORα4 and RORc2 have been shown to be significantly increased (Reppert et al., 2011). In addition to that, IL-17A is suggested to be responsible for an enhanced production of VEGF-C resulting in increased lymphangiogenesis. Finally, IL-17A expression also correlates with poor prognosis in NSCLC patients (Figure 3; Chen et al., 2010).

One explanation for the putative, tumor-promoting function of IL-17A is that the expression and the function of this cytokine are closely related to the proto-oncogene Stat3. On the one hand Stat3 regulates the expression of IL-17A whereas on the other hand IL-17A signaling in turn leads to an IL-6-dependent activation of Stat3 itself creating a positive feedback loop (Wang et al., 2009). Stat3 is known to have tumor-promoting properties as its expression in tumor cells has been associated with enhanced tumor cell survival, proliferation, and angiogenesis as well as with an accumulation of Tregs and myeloid-derived suppressor cells. Stat3 activates the expression of the anti-apoptotic gene Bcl-X_L_ as well as of IL-23, which has been reported to promote carcinogenesis also. Besides that, Stat3 inhibits the expression of IL-12 which is involved in anti-tumor responses via NK cell activation and Th1 induction (Hatton and Weaver, 2009; Wang et al., 2009). Consistent with this, IL-17A-deficient C57/Bl6 mice are characterized by a reduced Stat3 activation, as well as increased numbers of tumor infiltrating CD4^+^ and CD8^+^ T cells, which produce higher amounts of IFN-γ as compared to wild-type littermates. As a consequence, those IL-17A-deficient mice showed a reduced tumor growth rate in case of B16 melanoma as well as in case of MB49 bladder carcinoma (Wang et al., 2009).

Interestingly, the differentiation of Th17 cells depends on the concomitant action of IL-6 and the suppressive cytokine TGF-β which is also necessary for the induction of Tregs. IL-6, in turn, inhibits the development of Tregs suggesting that the differentiation of Tregs and Th17 cells could be mutually exclusive (Weaver et al., 2007). In contrast to this assumption, recent studies indicate that the differentiation of Th17 cells may even be connected to the development of Tregs (Zhou et al., 2009; Reppert et al., 2011). Thus, an up-regulation of Th17 cell lineage transcription factors has been shown to correlate with increased Foxp3 expression in patients with lung Ad. Moreover, blocking of IL-17A in a mouse model of lung Ad resulted in a decrease of Foxp3^+^ Treg numbers. This was accompanied by decreased levels of IL-6 and TGF-β, increased numbers of IFN-γ and TNF-α producing CD4^+^ T cells as well as a significant reduction of tumor growth (Reppert et al., 2011). As a conclusion these findings strongly support the idea that IL-17A is involved in tumor growth promotion. Therefore, anti-IL-17A treatment strategies could provide an attractive approach to lung cancer therapy.

## Conclusion

### Allergic asthma

Allergic asthma is characterized by a lack of Tregs and a pathological expansion of Th2 cells. Furthermore, it is suggested that there is an imbalance between Th1 and Th2 cells because under normal circumstances Th1 cells are thought to have a regulatory influence on Th2 cells. In addition, Th17 cells and especially their main cytokine IL-17A seem to play a significant role in the pathogenesis of asthma. Up to now, there is no therapy available to cure this disease. Airway tolerance is thought to provide the possibility to protect against and control the occurrence of asthma.

Transforming growth factor β is a pro- and anti-inflammatory cytokine which is up-regulated in the BALF of asthmatic patients. On the one hand it promotes airway remodeling and increases the mucus production leading to an exacerbation of asthma. On the other hand TGF-β, released by and acting on T regulatory cells, has an immunoregulatory function. The cytokine is able to suppress the proliferation of T cells and macrophages and therefore results in suppression of allergic asthma. Thus, the induction of TGF-β producing Tregs may represent a promising treatment in allergic asthma.

Interleukin-10, an anti-inflammatory cytokine, was found to be reduced in asthmatic patients. Simultaneously, Th2 cytokines were up-regulated in these patients suggesting that IL-10 has a suppressive effect on Th2 cells. There is increasing evidence that IL-10 and Tr1 cells are essential for the development of airway tolerance. The application of IL-10 as therapy has already been investigated in diseases such as psoriasis or rheumatoid arthritis. In this first clinical studies the data leads to the suggestion that IL-10 application is rather adequate to prevent than to cure psoriasis (Asadullah et al., 2003). These observations might also be true for allergic asthma. But the role of IL-10 in allergic asthma and therefore new treatment possibilities needs to be further elucidated.

Interleukin-17A is a pro-inflammatory cytokine which is up-regulated in the lung of asthmatic patients. It seems to be linked to neutrophilic and eosinophilic inflammation and perhaps also to AHR. Therefore, it can be speculated that targeting IL-17A is promising in asthma treatment. Investigators could already demonstrate that blocking IL-17A *per se* or upstream regulators and downstream messengers leads to reduced eosinophils, neutrophils, or Th2 cytokines. However, human studies are needed to gain deeper insight into the immunological and pathogenic role of IL-17A in allergic asthma.

### Lung adenocarcinoma

Regulatory T cell numbers are found to be increased in lung cancer patients which has been associated with poor prognosis. Thus, Tregs are thought to represent a predominant obstacle for the induction of anti-tumor immune responses and lung cancer therapy. Efficient anti-tumor immunity strongly depends on IFN-γ producing Th1 cells, mediating the activation of tumor-specific CD8 + CTLs which are required for the elimination of cancer cells. However, Tregs are suggested to antagonize these inflammatory effector cell responses (Byrne et al., 2011). It is possible that tumor cells contribute to the induction of Treg differentiation or at least to the recruitment of Tregs to the tumor site. Although the potential underlying mechanisms are not identified yet, it has been reported that several tumor types, including lung tumors, are able to produce considerable amounts of certain cytokines such as TGF-β, IL-10, or IL-17A. Interestingly, these cytokines are suggested to be connected to Treg development or function.

For instance, the immunosuppressive factor TGF-β is required for the development of iTregs. However, its role for tumor immunity is equivocal. Thus, it seems to show tumor suppressor activity at early stages of tumor development. However, in the course of tumor progression, cancer cells become insensitive to TGF-β mediated suppression. As a consequence, TGF-β acts as a tumor promoter in late-stage disease, probably due to its ability to induce iTregs and to suppress the differentiation of naïve T cells into effector T cells.

Another suppressive molecule, which is thought to be linked to Treg function, is IL-10. This cytokine is necessary for the development of T regulatory 1 cells (Tr1) and is involved in the regulation of TGF-β responses. NSCLC patients show significantly elevated IL-10 mRNA and serum levels as compared to healthy controls, which has been shown to be associated with poorer prognosis. However, data concerning the role of IL-10 for tumor progression are also partially inconsistent. Thus, some preclinical and clinical studies suggest that IL-10 might be important for tumor rejection, meaning that the role of IL-10 for lung cancer still needs to be elucidated.

The last molecule discussed in this review is IL-17A, which generally is a pro-inflammatory cytokine. However, in contrast to former assumptions, the development of IL-17-producing Th17 cells and Tregs is not mutually exclusive, but rather seems to be connected. According to this, blocking of IL-17A in a wild-type mouse model of lung Ad resulted in a decreased number of Foxp3^+^ Tregs and an increase of IFN-γ and TNF-α producing CD4^+^ T cells leading to a significant reduction of tumor growth.

As a conclusion, the suppression of Tregs by blocking the signaling pathways of particular cytokines may represent a promising approach to future lung cancer therapy.

## Conflict of Interest Statement

The authors declare that the research was conducted in the absence of any commercial or financial relationships that could be construed as a potential conflict of interest.

## Abbreviations

AHR, airway hyper-responsiveness; APC, antigen presenting cell; BAL, bronchoalveolar lavage; BALF, bronchoalveolar lavage fluid; CCL11, Chemokine (C–C motif) ligand 11; CXCR5, chemokine (C-X-C motif) receptor 5; DC, dendritic cell; ERK, extracellular signal-regulated kinase; FEV, forced expiratory volume; Foxp3, forkhead box protein 3; GSK-3β, glycogen synthase kinse-3β; hMPV, human meta-pneumovirus; ICOS/ICOS-L, inducible T cell co-stimulator/-ligand; IFN-γ, interferon-γ; IL, interleukin; iTreg, induced regulatory T cells; JAK1, Janus kinase; MAP, mitogen-activated protein; MCh, Methacholine; MHC, major histocompatibility complex; nat Treg, natural regulatory T cell; NSCLC, non-small cell lung cancer; OVA, Ovalbumin; pDC, plasmacytoid dendritic cell; PI3K, phosphatidylinositol 3-kinase; RSV, respiratory syncytial virus; RV, Rhinovirus; TAK1, transforming growth factor activated kinase; TAM, tumor-associated macrophage; TGF-β, transforming growth factor β; TGF-β R, transforming growth factor β receptor; Th, T helper cell; TIL, tumor infiltrating lymphocytes; TNF-α, tumor necrosis factor α; Tr1/3, regulatory T cells type 1/3; TRAF, (TNF)-receptor-associated factor; Treg, regulatory T cells.
